# Dementia clinic in general hospital settings

**DOI:** 10.4103/0019-5545.44904

**Published:** 2009

**Authors:** K. S. Shaji, Thomas Iype, K. Praveenlal

**Affiliations:** Department of Psychiatry, Medical College, Thrissur - 680596, Kerala, India; 1 Department of Neurology, Medical College, Thiruvanatha Puram, Kerala, India; 2 Principal, Medical College, Thrissur - 680596, Kerala, India

**Keywords:** Carers, dementia, developing countries, liaison psychiatry

## Abstract

**Background:**

There is a need to develop specialised dementia care services in developing countries.

**Materials and Methods:**

We used the existing infrastructure of a general hospital to start a weekly dementia clinic.

**Results:**

We were able to support home-based care, even in patients with advanced disease. This new service gave us opportunity to train clinicians and researchers interested in dementia.

**Conclusion:**

It is feasible to start weekly dementia clinics using existing infrastructure in developing countries. Networking of such centres can generate a database capable of guiding service development.

## INTRODUCTION

Dementia is an emerging public health problem in developing countries. However, it remains a hidden problem due to low levels of public awareness. There are no specialized services for older people in most of these countries. General practitioners are not trained to identify and manage dementia as the undergraduate medical curriculum has very little emphasis on geriatrics and dementia.

We started a monthly dementia clinic at the local primary health centre in October 1999 to provide services for cases identified during research undertaken by the 10/66 Dementia Research Group.[1–3] However, such services may be more feasible in general hospitals than in primary care settings. Clinicians with expertise in dementia care are mostly attached to general hospitals, which are often located in urban areas. The public awareness about dementia may also be better among the more literate people living in the urban areas.

So we started a new dementia care service in a general hospital using the existing infrastructure. This paper describes socio demographic and clinical details of the patients who availed this new out patient service and our experience in running it. The assessment and management of these patients will also be described.

## MATERIALS AND METHODS

We started a weekly Dementia Clinic at Medical College, Thrissur, in the South Indian state of Kerala in April 2002. Consultants from the department of neurology (TI) and psychiatry (SKS) lead the service. Diagnosis of dementia was made according to DSM IV criteria.[4] Additional diagnostic criteria were used for diagnosing dementia with Lewy bodies (DLB),[5] and frontotemporal dementia (FTD).[6] A trained clinician, administered instruments like Mini Mental State Examination (MMSE),[7] Everyday Abilities Scale for India (EASI),[8] Clinical Dementia Rating scale (CDR)[9] and Neuropsychiatric Inventory (NPI)[10] to all patients. Following this initial assessment, we gave the caregiver the option of bringing the patient to the clinic for follow up or to come without the patient. The main aim of Dementia Clinic was to support home-based care. The follow up data of patients recruited in the year 2004 was examined to assess the acceptance of this new service.

## RESULTS

The study recruited all new cases attending the dementia clinic at Medical College, Thrissur, from April 2002 to February 2004. A total of 202 patients were screened during this period and 137 of them (78 males, 59 women) received a diagnosis of dementia. Their mean age was 67.4 years (range 38 to 89), and the mean age of onset of dementia was 65.6 years.

The main subtypes of dementia were Alzheimer's disease (36.5%), Vascular Dementia (27%) and Dementia with Lewy Bodies (9.5%). Eleven patients (8%) had treatable causes. There were two cases each of meningioma, vitamin B 12 deficiency, neurosyphilis, normal pressure hydrocephalus and one case of subdural haematoma.

Fifty-eight (42.3%) patients had the onset of dementia before the age of 65 years. The main subtypes in both pre-senile and senile onset subgroups are given in Table 1. A quarter of patients in both the groups had Vascular Dementia.

**Table 1:** The main causes of dementia among the presenile onset and senile onset dementia cases

Cause	Presenile onset (%)	Senile onset (%)
Alzheimer's disease	13 (22.4)	37 (46.8)
Frontotemporal dementia	11 (19)	2 (2.5)
Dementia of Lewy bodies	3 (5.2)	7 (8.9)
Vascular dementia	15 (25.9)	22 (27.9)
Others	16 (27.6)	11 (13.9)
Total	58	79

Caregivers and family members took part in out patient sessions providing information and education about dementia. We trained caregivers to expect, anticipate and report symptoms. Patients with BPSD were particularly considered for pharmacological and or non-pharmacological interventions. Patients and/or their caregiver were followed up at regular intervals. This weekly clinic functioned as a drop-in clinic, where they could come earlier for follow up, if needed.

The patient/caregiver follow up improved over years. Those patients who came for follow up had a mean follow up of 207 days (range 4 to 461 days). Fifty percent of the patients recruited (51/102) had a follow of more than 90 days. All our patients were cared at home by the caregiver.

Dementia clinic provided opportunities for training undergraduate students and doctors undergoing postgraduate training in internal medicine. It also gave us opportunity to train researchers in the use of instruments like Geriatric Mental State examination,[11] Community Screening Instrument for Dementia,[12] NPI and CDR.

## DISCUSSION

We are now providing specialized dementia care services through the weekly dementia clinic. Though clinic based, this new service is able to support home-based care to a great extent. Even when patients are unable to come for follow up visits, the caregivers are encouraged to attend the clinic. This allows the specialist inputs to continue even during late stages of dementia. Out-patient dementia care services in developing countries will have to take up this extended responsibility of supporting home-based care of people with advanced disease. This is important, as institutional care is neither available nor affordable to most families. The need for such a service was evident from the eagerness of caregivers to make use of this new service.

Dementia care is in its infancy in most developing countries. Development of specialized services such as dementia clinics in general hospitals can be a good starting point. Such services should also aim at generating a useful database, capable of guiding service development. Dementia clinics can provide a setting, wherein clinicians and researchers interested in dementia can undergo further training. There is a need for many such centres to train clinicians in dementia care.

Availability of trained manpower makes detailed assessments possible. We identified many subtypes of dementia using standard definitions. The frequency of vascular dementia and other reversible causes of dementia merits further study. Reversible and treatable dementias may indeed be more prevalent in low-income countries. Future research needs to address this possibility.

Networking of these centres will allow exchange of useful information between clinicians engaged in dementia care. Geriatric Psychiatry speciality section of Indian Psychiatric Society is in the process of setting up a National Network of Dementia Clinics in India. This national network is expected to facilitate dementia research and guide service development in the country.

## CONCLUSION

It is feasible to start specialized dementia care services in general hospitals utilizing existing infrastructure. With caregivers as partners in care, such clinic-based services can extend considerable support to home-based care, even in the late stages of the illness.
